# Mortality and potential years of life lost due to COVID-19 among healthcare workers in Bahia, 2020-2022

**DOI:** 10.1590/S2237-96222025v34e20240315.en

**Published:** 2025-09-08

**Authors:** Renata Barbosa Vilaça Marques de Carvalho, Kionna Oliveira Bernardes Santos, Rita de Cássia Pereira Fernandes, Verônica Maria Cadena Lima, Luciane Gabriele Pereira Gomes Lopes, Rafaela Cordeiro Freire

**Affiliations:** 1 Universidade Federal da Bahia, Programa de Pós-Graduação em Saúde, Ambiente e Trabalho, Salvador, BA, Brazil

**Keywords:** COVID-19, Healthcare Personnel, Mortality, Life Expectancy, Descriptive Epidemiology, Covid-19, Personal de Salud, Mortalidad, Esperanza de Vida, Epidemiología Descriptiva

## Abstract

**Objective:**

Estimate mortality indicators and impact of COVID-19 on healthcare workers in Bahia in the period 2020-2022.

**Methods:**

This is a descriptive study, with death data extracted from the Brazilian Mortality Information System. Population data were obtained from professional councils, the National Registry of Health Establishments and the Brazilian National Immunization Program Information System. The mortality rate was calculated and presented per 1,000 workers. Potential years of life lost and productive years of life lost due to COVID-19 were estimated. An analysis was carried out between mortality rates and the proportion of people vaccinated against COVID-19.

**Results:**

Of the total number of deaths (n=403), the majority were female (63.3%), aged 40 or over (85.4%), of brown race/skin color (52.1%) and with secondary or technical education (48.1%). The highest mortality rates were observed among elderly/healthcare caregivers, health agents, veterinarians/zootechnicians, biologists and pharmacy/office attendants. Regarding the evolution of mortality, three waves were observed, with a reduction in deaths after the start of vaccination. In total, 6,771 potential years of life lost and 6,778 productive years of life lost were estimated, with a greater impact on women, in the 40-49 age group and in occupations that involved direct care.

**Conclusion:**

The results showed the potential impact generated by COVID-19 on the healthcare workers in Bahia.

## Resumen

**Objetivo:**

Estimar los indicadores de mortalidad y el impacto de la covid-19 en los trabajadores de la salud en Bahía en el período 2020-2022.

**Métodos:**

Se trata de un estudio descriptivo, con datos de defunciones extraídos del Sistema de Información de Mortalidad. Los datos de población se obtuvieron de los consejos profesionales, del Registro Nacional de Establecimientos de Salud y del Sistema de Información del Programa Nacional de Inmunizaciones. Se calculó y presentó la tasa de mortalidad por cada 1.000 trabajadores. Se estimaron los años de vida potencial perdidos y los años de vida productiva perdidos debido al covid-19. Se realizó un análisis entre las tasas de mortalidad y la proporción de personas vacunadas contra el covid-19.

**Resultados:**

Del total de fallecidos (n=403), la mayoría fueron de sexo femenino (63,3%), de 40 años o más (85,4%), de raza/color de piel pardo (52,1%) y con escolaridad secundaria o técnica (48,1%). Las tasas de mortalidad más altas se observaron entre los cuidadores de ancianos/sanitarios, agentes de salud, veterinarios/zootecnistas, biólogos y asistentes de farmacia/consultorio. En cuanto a la evolución de la mortalidad, se observaron tres olas, con una reducción de las muertes tras el inicio de la vacunación. En total, se estimaron 6.771 años potenciales de vida perdidos y 6.778 años de vida productiva perdidos, con mayor impacto en las mujeres, en el grupo de edad de 40 a 49 años y en ocupaciones que implicaban cuidado directo.

**Conclusión:**

Los resultados mostraron el impacto potencial generado por la covid-19 en la fuerza laboral de salud en Bahía.

**Ethical aspects**

This research used public domain anonymized databases.

## Introduction

A virus, called Severe Acute Respiratory Syndrome Coronavirus 2 (SARS-CoV-2), was first identified in Wuhan, China, in December 2019 (1,2). On January 30, 2020, the disease caused by this virus, COVID-19, was declared a Public Health Emergency of International Concern and, on March 11 of the same year, as a pandemic, due to the increase in the number of cases and the rapid spread of the virus to multiple countries on different continents (3,4).

Brazil led the top positions in the number of cases and deaths in the world ranking, coming in fifth place in the number of cases and second in the number of deaths, behind only the United States and India in the number of deaths (5). 

At the beginning of the pandemic, data on cases and deaths from COVID-19 in Brazil was restricted to healthcare professionals and public safety workers. In the second half of 2020, there was an expansion to all occupational categories. This variable is no longer mandatory, which has led to under-registration. In Brazil, among health professionals, the highest number of cases of COVID-19 was recorded in nursing professionals, followed by doctors and community health agents (6). In Bahia, most cases also occurred in nursing professionals, doctors and community health agents (7). 

During the COVID-19 pandemic, several individual and community measures were adopted to minimize the spread of the virus and morbidity and mortality due to the absence of specific treatment and vaccine against SARS-CoV-2 (8). In Brazil, there was a lack of central coordination in combating the pandemic and timely implementation of immunization against COVID-19 (9). 

The Brazilian vaccination campaign against COVID-19 began in Brazil in January 2021, with healthcare workers as a priority group. After the introduction of vaccination, there was a decrease in the number of serious cases and deaths (8). 

As of March 2023, the country ranked fifth in the world in terms of the absolute number of people who received at least the first dose of the COVID-19 vaccine, corresponding to 89.10% of the vaccinated population (10). On May 5, 2023, due to the improvement in the epidemiological scenario and the progress of vaccination against COVID-19, the end of the Public Health Emergency of International Importance regarding COVID-19 was declared (11). 

In the context of the COVID-19 pandemic, the poor quality of data and the lack of adequate coverage of information systems on workers health in Brazil became evident. The lack of data on some occupations limited and hindered the work of health surveillance in planning actions aimed at protecting workers. This gap made it impossible to infer work as a determining factor for illness. Another important limitation corresponded to the difficulty of calculating coefficients by occupation due to the lack of data on the exposed worker population to compose the denominator (12).

Considering the social and economic impact of premature mortality among healthcare workers and the shortage of studies that assess mortality from COVID-19 in different groups of health workers, due to the underreporting of the occupation variable in information systems and Bahia’s pioneering role in developing actions in worker health surveillance (13), it becomes extremely important to measure the magnitude of mortality of healthcare workers. Therefore, the question is: What was the impact of COVID-19 mortality on different groups of healthcare workers in Bahia?

This study aimed to estimate mortality indicators and impact of COVID-19 on different groups of healthcare workers in Bahia in the period 2020-2022.

## Methods

### Study design

This was a descriptive study of mortality indicators and impact of COVID-19 on healthcare workers in Bahia, between March 2020 and December 2022.

### Context

Bahia is a Brazilian state located in the Northeast region, with a territorial extension of 564,760.429 km^2^ and a population density of 25.04 inhabitants/km^2^, being considered the fourth most populated state in the country, with an estimated population of 14,850,513 inhabitants in 2024. The state is made up of 417 municipalities, with Salvador as its capital. Its territory is divided into nine health macro-regions (Central-East, Central-North, Extreme South, East, Northeast, North, West, Southeast and South), subdivided into 28 health regions (14,15).

According to data from 2025, Bahia has 22,686 public and private health establishments, of which 4,963 are basic care units, 132 emergency and urgency units, 517 general hospitals, 72 specialized hospitals, 26 mixed units, 156 day hospitals, two central public health laboratories and 58 public health laboratories. There are 11,748 clinical beds, with 83.7% of the beds available for the Brazilian Unified Health System (SUS) and 1,642 beds in intensive care units. It also has 16 reference centers for workers health (16).

### Participants 

Only healthcare sector workers participated in the study, that is, occupations were selected according to the Brazilian Classification of Occupations grouping that was directly linked to the healthcare sector. Subsequently, the occupational groups were consolidated into distinct occupational categories.

### Variables 

The following variables were considered: sex, age group, race/skin color, level of education and occupation.

### Data sources and measurement

Data on the number of deaths were extracted from the Mortality Information System. This data was requested from the Health Department of the State of Bahia and made available as microdata by the Information Technology Department of the Unified Health System (https://datasus.saude.gov.br/transferencia-de-arquivos/#). Deaths from COVID-19 were considered to be all records with ICD B34.2 (Infection by Coronavirus of unspecified location) followed by codes U07.1 (COVID-19, laboratory-confirmed case) or U07.2 (Covid-19, unidentified virus, clinical-epidemiological), which were the markers of the pandemic in Brazil, defined by the World Health Organization, in part 1 of the death certificate, regardless of their position in the chain of causes. 

In an attempt to overcome the underestimation of healthcare professionals, different sources of information were used to estimate the exposed population with a view to constructing mortality coefficients. The data came from professional councils, the National Registry of Health Establishments (http://cnes.datasus.gov.br/) and the Brazilian National Immunization Program Information System (https://opendatasus.saude.gov.br/dataset/covid-19-vaccination). For the latter, the number of healthcare workers in Bahia vaccinated with the first dose against COVID-19 was considered. 

To calculate the mortality rate, the number of deaths from COVID-19 among healthcare workers was used in the numerator. In the denominator, to quantify the population exposed to the risk of dying from this disease, the populations reported by the data sources used in this study were used, when available. A comparison was made between these three sources of population information to verify the consistency of this indicator. The values calculated for the mortality coefficient were presented per 1,000 workers.

To verify the effect of deaths occurring early in relation to the length of life for the study population, the potential years of life lost and productive years of life lost due to COVID-19 were estimated, per individual, in each age group. The mathematical formula proposed by Romeder and McWhinnie (17) was used, adopting 70 years as the upper age limit.

The age at which the worker died from COVID-19 was subtracted from the upper age limit adopted (representing life expectancy) to estimate the potential years of life lost. The result was multiplied by the number of deaths from COVID-19 that occurred in the same age group. The values obtained for all workers who died in each age group were added together.

To calculate the productive years of life lost, the midpoint of the age class interval was considered, plus a correction factor of 0.5 (used when it is assumed that all deaths occurred in the middle of the year). This value was subtracted from the upper age limit adopted as the cut-off point. The result of this subtraction was multiplied by the number of deaths from COVID-19 that occurred in the same age group. The values obtained were added for each age group, generating the total number of productive years of life lost.

The mortality coefficient for COVID-19 and the proportion of people vaccinated for the disease among healthcare workers in Bahia were analyzed, by four-month period, in order to verify the trend in the mortality curve. National Registry of Health EstablishmentsTo calculate the proportion of vaccinated people, the number of healthcare workers vaccinated in the period was used in the numerator and, in the denominator, the average per four-month period of healthcare workers included in the National Registry of Health Establishments.

### Statistical methods

Absolute and relative frequencies were used for categorical variables. Mean and standard deviation were calculated for continuous variables. Data were tabulated in Microsoft Excel 16.0 and analyzed in SPSS 20.0. 

## Results

During the period analyzed, 17,605 deaths from COVID-19 were reported in Bahia, of which 4,081 (23.2%) had no occupation, 2,247 (12.8%) were retirees and pensioners, 384 (2.2%) were unemployed, 403 (2.3%) were healthcare workers and 8,933 (50.7%) were other workers. 

Of the total number of healthcare workers who died from COVID-19, the majority were female (63.3%), aged 40 or over (85.4%), of brown race/skin color (52.1%) and with secondary or technical education (48.1%) (Table 1). The mean age was 53.2 years, with a standard deviation of 10.9 years.

**Table 1:** Sociodemographic characteristics of healthcare workers who died from COVID-19. Bahia, 2020-2022 (n=403)

Variables	n (%)
Gender	
Female	255 (63.3)
Male	148 (36.7)
**Age group** (years)	
19-29	6 (1.5)
30-39	53 (13.1)
40-49	94 (23.3)
50-59	105 (26.1)
60-69	145 (36.0)
**Race/skin color**	
White	112 (27.8)
Black	68 (16.9)
Brown	210 (52.1)
Ignored/blank	13 (3.2)
Education	
Low education level	51 (12.7)
Intermediate and technical level	194 (48.1)
Higher education	143 (35.5)
Ignored/Blank	15 (3.7)

The occupations with the highest number of deaths were nursing technicians/assistants (29.0%), health agents (13.4%) and elderly caregivers/healthcare caregivers (10.4%). The general mortality rate from COVID-19 among healthcare workers in Bahia was similar among the three sources of information used to compose the population exposed to death from COVID-19. There was a discrepancy between the mortality coefficients by occupation, but there was agreement between the three sources of information regarding the occupations of veterinarians/zootechnicians and biologists, as they presented one of the highest mortality coefficients. The mortality rates, calculated based on population data from the National Registry of Health Facilities (CNES) and the Information System of the National Immunization Program (SI-PNI), showed concordance regarding the highest values observed among elderly/health caregivers, but diverged with respect to community health agents and pharmacy/clinic attendants (Table 2).

**Table 2:** Deaths, population extracted from the National Registry of Health Establishments (CNES), council and Brazilian National Immunization Program Information System (SI-PNI), and mortality coefficient due to COVID-19, according to occupation, in healthcare workers. Bahia, 2020-2022 (n=403)

Occupations	Deaths	Population			Mortality coefficient		
	n (%)	CNES	Council	SI-PNI	CNES	Council	SI-PNI
Health agents	54 (13.4)	39,348	..	1,931	1.4	..	28.0
Social workers	13 (3.2)	2,759	18,499	4,298	4.7	0.7	3.0
Pharmacy/office workers	29 (7.2)	1,990	..	...	14.6	..	..
Biologists	4 (1.0)	242	3,260	349	16.5	1.2	11.5
Biomedical	0 (0.0)	851	...	2,071	..	..	..
Dental surgeons	12 (3.0)	9,673	15,520	11,809	1.2	0.8	1.0
Ambulance drivers	0 (0.0)	2,382	..	12,497	0.0	..	..
Elderly/Health Caregivers	42 (10,4)	236	..	8,284	178.0	..	5.1
Physical educators	4 (1.0)	608	...	2.611	6.6	...	1.5
Nurses	31 (7.7)	23,066	39,675	32,766	1.3	0.8	0.9
Pharmacists	10 (2.5)	3,817	11,045	8,828	2.6	0.9	1.1
Physiotherapists/Chiropractors	6 (1.5)	6,468	17,163	12,672	0.9	0.3	0.5
Speech therapists	0 (0,0)	1,089	1,850	1,332	..	..	..
Health service managers	1 (0.2)	2,229	..	...	0.4	..	..
Physicians	30 (7,4)	33,542	26,192	21,508	0.9	1.1	1.4
Nutritionists	1 (0.2)	2,585	10,446	5,739	0.4	0.1	0.2
Others^a^	9 (2.2)	306	..	1,676	29.4	..	5.4
Psychologists/ psychoanalysts	14 (3,5)	3,831	17,696	7,478	3.7	0.8	1.9
Nursing technicians/ assistants	117 (29.0)	47,870	99,200	62,948	2.4	1,2	1.9
Nursing technicians/ laboratory assistants/Blood bank assistants	12 (3.0)	4,068	..	...	2.9	..	..
Nursing technicians/ assistants	5 (1.2)	4,745	..	4,423	1.1	..	1.1
Radiology technologists/ technicians	1 (0.2)	2,689	..	...	0.4	...	...
Occupational therapists/ orthoptists	1 (0.2)	370	624	425	2.7	1.6	2.4
Veterinarians/ Zootechnicians	7 (1.7)	264	5,590	1,968	26.5	1.3	3.6
Total	403 (100.0)	195,026	266,760	205,613	2.1	1.5	2.0

^a^Doulas, beauticians, massage therapists, podiatrists, acupuncture technicians, chiropractic technicians, holistic therapists, optician and optometry technicians, maintenance technicians of medical-hospital equipment and instruments, orthopedic technicians, orthopedic immobilization technicians and nutrition and dietetics technicians.

**Figure 1:**
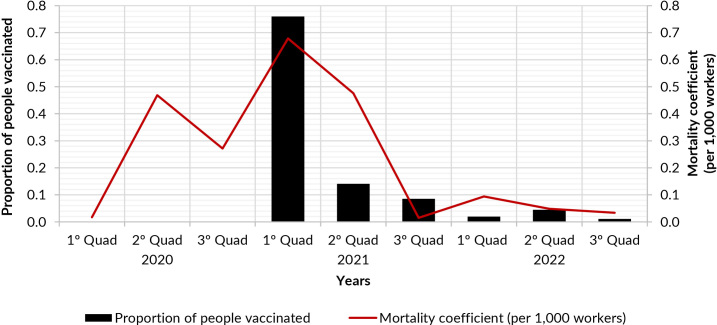
Mortality coefficient and proportion of people vaccinated for COVID-19 among healthcare workers, by four-month period. Bahia, 2020-2022

In terms of the evolution of mortality from COVID-19 in health workers, there was an increase in mortality in the second four months of 2020. Subsequently, there was a reduction in mortality, which rose again in the first four months of 2021. From the second quarter of 2021, there was a sharp drop in mortality. There was a high concentration of workers vaccinated with the first dose of the COVID-19 vaccine in the first four months of 2021, with a reduction in the following months (Figure 1).

An estimated 6,771.0 potential years of life lost and 6,778.0 productive years of life lost were estimated, of which 4,220.0 potential years of life lost and 4,186.0 productive years of life lost were among females, corresponding to 62.3% and 61.8% of the loss. The loss was greater in females in all age groups except those aged 19-29. The greatest impact on the number of potential years of life lost and productive years of life lost was observed in the age group of 40-49 years, totaling 34.2% (2,317.0 potential years of life lost) and 34.7% (2,350.0 productive years of life lost) of the loss. The smallest impact was observed in the 19-29 age group (263.0 potential years of life lost; 273.0 productive years of life lost). The year 2021 brought a loss of 56.2% (3,804.0 potential years of life lost) and 55.9% (3,786.5 productive years of life lost) (Table 3). In relation to the average of potential years of life lost/death, each deceased woman lost 16.5 years and each man, 17.2 years. 

**Table 3:** Potential years of life lost (PYLL) and Productive years of life lost (PrYLL) due to COVID-19 in healthcare workers, by age group and sex. Bahia, 2020-2022 (n=403)

Age group (years)	Gender	2020		2021		2022		Total	
		PYLL	PrYLL	PYLL	PrYLL	PYLL	PrYLL	PYLL	PrYLL
19-29	Female	43.0	45.5	0.0	0.0	51.0	45.5	94.0	91.0
	Male	0.0	0.0	126.0	136.5	43.0	45.5	169.0	182.0
	Total	43.0	45.5	126.0	136.5	94.0	91.0	263.0	273.0
30-39	Female	340.0	350.0	580.0	595.0	136.0	140.0	1,056.0	1,085.0
	Male	405.0	420.0	344.0	350.0	0.0	0.0	749.0	770.0
	Total	745.0	770.0	924.0	945.0	136.0	140.0	1,805.0	1,855.0
40-49	Female	493.0	500.0	964.0	975.0	46.0	50,0	1,503.0	1,525.0
	Male	239.0	250.0	520.0	525.0	55.0	50,0	814.0	825.0
	Total	732.0	750.0	1,484.0	1,500.0	101.0	100.0	2,317.0	2,350.0
50-59	Female	499.0	480.0	476.0	480.0	68.0	60.0	1,043.0	1,020.0
	Male	229.0	240.0	295.0	300.0	15.0	15.0	539.0	555.0
	Total	728.0	720.0	771.0	780.0	83.0	75.0	1,582.0	1,575.0
60-69	Female	133.0	125.0	312.0	270.0	79.0	70.0	524.0	465.0
	Male	57.0	70.0	187.0	155.0	36.0	35.0	280.0	260.0
	Total	190.0	195.0	499.0	425.0	115.0	105.0	804.0	725.0
Total	Female	1,508.0	1,500.5	2,332.0	2,320.0	380.0	365.5	4,220.0	4,186.0
	Male	930.0	980.0	1,472.0	1,466.5	149.0	145.5	2,551.0	2,592.0
	Total	2,438.0	2,480.5	3,804.0	3,786.5	529.0	511.0	6,771.0	6,778.0

The occupations that presented the greatest losses were those that worked directly in assistance care, that is, who were more exposed to illness from COVID-19 (Table 4). The healthcare workers affected early, between 19-29 years old, were those with secondary and technical education, dentists, physical educators and psychologists/psychoanalysts. In the age group of 40-49 years, the occupations with the greatest loss were workers with secondary and technical education, nurses and doctors (Table 4).

**Table 4:** Potential years of life lost (PYLL) and Productive years of life lost (PrYLL) due to COVID-19 in healthcare workers, by occupation and age group. Bahia, 2020-2022 (n=403)

Occupation	19-29 years old		30-39 years old		40-49 years old		50-59 years old		60-69 years old		Total	
	PYLL	PrYLL	PYLL	PrYLL	PYLL	PrYLL	PYLL	PrYLL	PYLL	PrYLL	PYLL	PrYLL
Health agents	0.0	0.0	229.0	245.0	317.0	325.0	222.0	225.0	117.0	95.0	885.0	890.0
Social workers	0.0	0.0	32.0	35.0	45.0	50,0	60.0	60.0	42.0	30.0	179.0	175.0
Pharmacy/medical office attendants	43.0	45.5	110.0	105.0	121.0	125.0	200.0	210.0	44.0	30.0	518.0	515.5
Biologists	0.0	0.0	0.0	0.0	51.0	50,0	0.0	0.0	8.0	10.0	59.0	60.0
Dental surgeons	43.0	45.5	77.0	70.0	22.0	25.0	60.0	60.0	7.0	20.0	209.0	220.5
Elderly/Health Caregivers	0.0	0.0	0.0	0.0	393.0	400.0	245.0	225.0	74.0	55.0	712.0	680.0
Physical educators	42.0	45.5	35.0	35.0	47.0	50,0	0.0	0.0	0.0	0.0	124.0	130.5
Nurses	0.0	0.0	301.0	315.0	206.0	200.0	94.0	90.0	38.0	40.0	639.0	645.0
Pharmacists	0.0	0.0	66.0	70.0	46.0	50,0	50,0	45.0	13.0	15.0	175.0	180.0
Physiotherapists/Chiropractors	0.0	0.0	141.0	140.0	0.0	0.0	0.0	0.0	15.0	10.0	156.0	150.0
Health service managers	0.0	0.0	0.0	0.0	0.0	0.0	16.0	15.0	0.0	0.0	16.0	15.0
Physicians	0.0	0.0	0.0	0.0	101.0	100.0	127.0	135.0	88.0	85.0	316.0	320.0
Nutritionists	0.0	0.0	0.0	0.0	0.0	0.0	20.0	15.0	0.0	0.0	20.0	15.0
Others^a^	0.0	0.0	101.0	105.0	72.0	75.0	0.0	0.0	14.0	15.0	187.0	195.0
Psychologists/psychoanalysts	41.0	45.5	101.0	105.0	79.0	75.0	17.0	15.0	16.0	30.0	254.0	270.5
Nursing technicians/assistants	43.0	45.5	442.0	455.0	695.0	700.0	421.0	420.0	255.0	235.0	1,856.0	1,855.5
Laboratory/Blood bank technicians/assistants	0.0	0.0	135.0	140.0	27.0	25.0	11.0	15.0	43.0	30.0	216.0	210.0
Oral health technicians/assistants	51.0	45.5	0.0	0.0	47.0	50,0	15.0	15.0	9.0	5.0	122.0	115.5
Radiology technologists/technicians	0.0	0.0	0.0	0.0	0.0	0.0	12.0	15.0	0.0	0.0	12.0	15.0
Occupational therapists/orthoptists	0.0	0.0	0.0	0.0	0.0	0.0	0.0	0.0	9.0	5.0	9.0	5.0
Veterinarians/zootechnicians	0.0	0.0	35.0	35.0	48.0	50,0	12.0	15.0	12.0	15.0	107.0	115.0
Total	263.0	273.0	1,805.0	1,855.0	2,317.0	2,350.0	1,582.0	1,575.0	804.0	725.0	6,771.0	6,778.0

^a^Pedologists, opticians, optometry technicians, orthopedic technicians, beauticians and masseurs.

## Discussion

This research estimated the impact of COVID-19 mortality on different groups of healthcare workers in Bahia. A greater impact was observed in women, in the age group of 40-49 years, among mixed race people, with secondary and technical education, and in the occupations of nursing technicians/assistants, health agents, elderly/health care workers, nurses, pharmacy/office attendants and doctors. The overall mortality rate for COVID-19 was similar across the three sources of information used, although there were discrepancies when comparing the coefficients by occupation. Regarding the evolution of mortality, three waves were observed, with markedly different magnitudes in the last year. The start of vaccination in the first four months of 2021 resulted in a drastic reduction in mortality among healthcare workers.

Male gender, advanced age and comorbidity are associated with a higher occurrence of deaths from COVID-19 in healthcare workers and non-healthcare workers (18-21). In this study, a greater number of deaths were observed in females. This is possibly due to the greater proportion of women in the health sector, given that it was not possible to calculate the mortality coefficient by sex, as there was no population data available on health workers. The highest proportion of deaths increased with age. 

The results of this study draw attention to the occupations of elderly/healthcare caregivers, veterinarians/zootechnicians, biologists and pharmacy/office attendants, as they had the highest mortality rates and until 2024 there were no reports in the literature on mortality from COVID-19 for these occupations. Given the fragility of the occupation variable, even in death records, and the profound divergences in the denominators, these findings should be highlighted, but the strength of the evidence should be taken with the necessary caution.

In Mexico, it was found that people working in ambulances (44.8/10,000 workers), physiotherapists (22.4/10,000 workers) and pharmacy teams (20.7/10,000 workers) had the highest mortality rates (21). Although the COVID-19 pandemic has shown stability and a reduction in severity and mortality, studies that have calculated the mortality rate among healthcare workers remain scarce in the literature, possibly due to the difficulty in obtaining data on the population exposed to the risk of dying from the disease.

Mortality from COVID-19 evolved in three waves, with the highest peak in the second, and the lowest coefficients recorded in the third (9), corroborating the findings of this study. The evolution of mortality in the second wave could be explained by the emergence of the new Delta variant (22), which coincided with the start of vaccination. Vaccination campaigns have reduced morbidity and mortality from COVID-19 (23,24). 

By surveying deaths, it was possible to estimate impact indicators, which revealed the social value of premature mortality and the reduction in the workforce. The findings of this study demonstrated a greater impact on young workers in the 40-49 age group. When death occurs at a stage in which life is potentially productive, it affects not only the individual, but also society as a whole, as its economic and intellectual potential is lost (25,26). 

The years of potential life lost is an indicator that measures how long a person would have lived if they had not died prematurely. This indicator makes it possible to identify the age groups most affected by the disease and has proven effective in guiding the prioritization of public health interventions (25-27).

Until 2024, there were no studies with the same methodological scope on potential years of life lost and productive years of life lost due to COVID-19 in healthcare workers, which made it impossible to make a comparison. In Brazil, the years of life lost due to premature death from COVID-19 were higher among male nurses and nursing technicians aged 41-50 (28). There was also a greater loss among nursing professionals in the 31-40 age group, with a lower loss among younger professionals (29). 

There was a major impact on the potential years of life lost due to COVID-19, considering race/skin color and gender. Greater loss was observed among men, and black and indigenous populations were more affected in younger age groups, compared to white and yellow populations (30).

This study had limitations that should be considered when interpreting the results. Firstly, secondary data were used which may interfere with the validity of the findings, with selection and information bias, especially due to the incompleteness and underreporting of the data, with no data on some occupations. Secondly, there was underestimated population data and a lack of data stratified by sex and age to compose the denominator. Third, a single upper age limit of 70 years was used to calculate potential years of life lost and productive years of life lost. This approach did not take into account that life expectancy varies with sex, which may lead to an overestimation of the measure for males, given that life expectancy in males is lower than in females. 

The strengths of this study included the diversity of categories of healthcare workers studied and the strategy adopted to overcome the absence of a single population registry of healthcare workers by searching for three sources: National Registry of Health Establishments, professional councils and Brazilian National Immunization Program Information System. This initiative allowed the calculation of mortality coefficients that have not been calculated in most studies identified with healthcare workers. It has highlighted the unprecedented nature of quantifying potential years of life lost and productive years of life lost due to COVID-19 in healthcare workers. 

The results of this study showed the potential impact generated by COVID-19 on the healthcare workforce in Bahia, especially in groups of workers little mentioned in the literature, even though they are linked to professional councils. The results brought contributions that can support intervention strategies in the field of workers health and provide information on the impact of COVID-19 on healthcare workers; serve as a learning experience for new public health emergencies; and promote public policies to combat epidemics of respiratory diseases, such as the vaccination program and actions aimed at promoting workers health. 

Despite efforts to understand the behavior of COVID-19, there are still unknown social and economic costs. Therefore, new studies on the magnitude of premature mortality among healthcare workers are needed, in the context of the COVID-19 pandemic. 

## Data Availability

The databases used in this study are publicly accessible, and the links to them are detailed in the methods. Tabulated data is available at: https://demo.dataverse.org/dataset.xhtml?persistentId=doi%3A10.70122%2FFK2%2FSYHSJL&version=DRAFT.
