# Supplementary material for: Mortality and potential years of life lost due to COVID-19 among healthcare workers in Bahia, 2020-2022
Source: Epidemiol Serv Saude. 2025 Sep 8;34:e20240315. doi: 10.1590/S2237-96222025v34e20240315.en (PMC12435958; doi:10.1590/S2237-96222025v34e20240315.en)
Supplement: Translation

# Translation: Mortalidade e anos potenciais de vida perdidos por covid-19 em trabalhadores da saúde na Bahia, 2020-2022

**DOI:** 10.1590/S2237-96222025v34e20240315.pt

**Authors:** Renata Barbosa Vilaça Marques de Carvalho, Kionna Oliveira Bernardes Santos, Rita de Cássia Pereira Fernandes, Verônica Maria Cadena Lima, Luciane Gabriele Pereira Gomes Lopes, Rafaela Cordeiro Freire

**Affiliations:** 1 Universidade Federal da Bahia, Programa de Pós-Graduação em Saúde, Ambiente e Trabalho, Salvador, BA, Brasil

**Keywords:** Covid-19, Pessoal de Saúde, Mortalidade, Expectativa de Vida, Epidemiologia Descritiva

## Abstract

**Objetivo:**

Estimar indicadores de mortalidade e impacto por covid-19 em trabalhadores da saúde na Bahia no período 2020-2022.

**Métodos:**

Trata-se de estudo descritivo, com dados de óbitos extraídos do Sistema de Informações sobre Mortalidade. Os dados populacionais foram obtidos dos conselhos profissionais, do Cadastro Nacional de Estabelecimentos de Saúde e do Sistema de Informações do Programa Nacional de Imunizações. O coeficiente de mortalidade foi calculado e apresentado por 1 mil trabalhadores. Anos potenciais de vida perdidos e anos produtivos de vida perdidos por covid-19 foram estimados. Foi realizada análise entre os coeficientes de mortalidade e proporção de vacinados contra covid-19.

**Resultados:**

Do total de óbitos (n=403), a maior parte era do sexo feminino (63,3%), com idade igual ou superior a 40 anos (85,4%), da raça/cor da pele parda (52,1%) e com ensino médio e técnico (48,1%). Os maiores coeficientes de mortalidade foram observados em cuidadores de idosos/em saúde, agentes de saúde, veterinários/zootecnistas, biólogos e atendentes de farmácia/consultório. Com relação à evolução da mortalidade, observaram-se três ondas, com redução dos óbitos após o início da vacinação. No total, foram estimados 6.771 anos potenciais de vida perdidos e 6.778 anos produtivos de vida perdidos, com maior impacto no sexo feminino, na faixa etária de 40-49 anos e nas ocupações que atuavam diretamente nos cuidados assistenciais.

**Conclusão:**

Os resultados mostraram potencial impacto gerado pela covid-19 na força de trabalhadores da saúde da Bahia.

**Aspectos éticos**

Esta pesquisa utilizou bancos de dados de domínio público e anonimizados.

## Introdução

Um vírus, denominado Coronavírus da Síndrome Respiratória Aguda Grave 2 (SARS-CoV-2), foi identificado pela primeira vez em Wuhan, na China, em dezembro de 2019 (1,2). Em 30 de janeiro de 2020, a doença ocasionada por esse vírus, a covid-19, foi declarada como Emergência de Saúde Pública de Importância Internacional e, em 11 de março do mesmo ano, como uma pandemia, devido ao aumento do número de casos e à rápida disseminação do vírus para diversos países em diferentes continentes (3,4).

O Brasil liderou as primeiras posições no número de casos e de óbitos no ranking mundial, ficando em quinto lugar no número de casos e em segundo no número de óbitos, atrás somente dos Estados Unidos e da Índia no número de óbitos (5). 

No início da pandemia, os dados sobre os casos e óbitos por covid-19 no Brasil eram restritos aos profissionais da saúde e trabalhadores de segurança pública. No segundo semestre de 2020, houve ampliação para todas as categorias ocupacionais. Essa variável não passou a ser de preenchimento obrigatório, o que favoreceu o subregistro. No Brasil, entre os profissionais da saúde, o maior número de casos por covid-19 foi registrado em profissionais da enfermagem, seguido por médicos e agentes comunitários de saúde (6). Na Bahia, a maioria dos casos também ocorreu em profissionais da enfermagem, médicos e agentes comunitários de saúde (7). 

Durante a pandemia da covid-19, várias medidas individuais e comunitárias foram adotadas para minimizar a propagação do vírus e a morbimortalidade devido à ausência de tratamento específico e vacina contra o SARS-CoV-2 (8). No Brasil, houve falta de coordenação central no combate à pandemia e implementação oportuna da imunização contra a covid-19 (9). 

A campanha nacional de vacinação contra a covid-19 foi iniciada no Brasil em janeiro de 2021, tendo os trabalhadores da saúde como grupo prioritário. Após a introdução da vacinação, houve diminuição do número de casos graves e mortes (8). 

Até março de 2023, o país ocupava o quinto lugar mundial em número absoluto de pessoas que receberam pelo menos a primeira dose da vacina contra a covid-19, correspondendo a 89,10% da população vacinada (10). Em 5 de maio de 2023, devido à melhora do cenário epidemiológico e ao avanço da vacinação contra a covid-19, foi declarado o fim da Emergência de Saúde Pública de Importância Internacional referente à covid-19 (11). 

No contexto da pandemia da covid-19, ficaram evidentes a má qualidade dos dados e a falta de cobertura adequada dos sistemas de informação sobre a saúde do trabalhador no Brasil. A ausência de dados em algumas ocupações limitou e dificultou a atuação da vigilância em saúde no planejamento de ações direcionadas para a proteção dos trabalhadores. Essa lacuna impossibilitou a inferência do trabalho como fator determinante para o adoecimento. Outra limitação importante correspondeu à dificuldade de calcular coeficientes por ocupação devido à ausência de dados sobre a população de trabalhadores exposta para compor o denominador (12).

Considerando o impacto social e econômico da mortalidade prematura entre trabalhadores da saúde e a escassez de estudos que avaliam a mortalidade por covid-19 em diferentes grupos de trabalhadores da saúde, devido ao subregistro da variável ocupação nos sistemas de informação e o pioneirismo da Bahia no desenvolvimento de ações em vigilância em saúde do trabalhador (13), torna-se de suma importância medir a magnitude da mortalidade em trabalhadores da saúde. Assim, questiona-se: Qual o impacto da mortalidade pela covid-19 em diferentes grupos de trabalhadores da saúde na Bahia?

Este estudo teve como objetivo estimar os indicadores de mortalidade e impacto pela covid-19 em diferentes grupos de trabalhadores da saúde na Bahia no período 2020-2022.

## Métodos

### Delineamento

Tratou-se de estudo descritivo dos indicadores de mortalidade e impacto por covid-19 em trabalhadores da saúde, na Bahia, entre março de 2020 e dezembro de 2022.

### Contexto

A Bahia é um estado brasileiro, localizado na região Nordeste, com extensão territorial de 564.760,429 km^2^ e densidade demográfica de 25,04 habitantes/km^2^, sendo considerado o quarto estado mais povoado do país, com população estimada de 14.850.513 habitantes em 2024. O estado é constituído por 417 municípios, sendo sua capital Salvador. O seu território é dividido em nove macrorregiões de saúde (Centro-Leste, Centro-Norte, Extremo Sul, Leste, Nordeste, Norte, Oeste, Sudeste e Sul), subdividido em 28 regiões de saúde (14,15).

Conforme dados de 2025, a Bahia tem, em seu território, 22.686 estabelecimentos de saúde, públicos e privados, sendo 4.963 unidades de atenção básica, 132 unidades de urgência e emergência, 517 hospitais gerais, 72 hospitais especializados, 26 unidades mistas, 156 hospitais-dia, dois laboratórios centrais de saúde pública e 58 laboratórios de saúde pública. Existem 11.748 leitos clínicos, com 83,7% dos leitos disponíveis para o Sistema Único de Saúde e 1.642 leitos em unidade de terapia intensiva habilitados. Conta também com 16 centros de referência em saúde do trabalhador (16).

### Participantes 

Participaram do estudo apenas trabalhadores do setor saúde, ou seja, foram selecionadas as ocupações pela família da Classificação Brasileira de Ocupações que estava ligada diretamente ao setor saúde. Posteriormente, as famílias de ocupações foram agrupadas em categorias ocupacionais únicas.

### Variáveis 

Foram consideradas as seguintes variáveis: sexo, faixa etária, raça/cor da pele, nível de escolaridade e ocupação.

### Fontes de dados e mensuração

Os dados sobre o número de óbitos foram extraídos do Sistema de Informações sobre Mortalidade. Esses dados foram solicitados à Secretaria da Saúde do Estado da Bahia e disponibilizados como microdados pelo Departamento de Informática do Sistema Único de Saúde (https://datasus.saude.gov.br/transferencia-de-arquivos/#). Foram considerados óbitos por covid-19 todo registro com a CID B34.2 (infecção por coronavírus de localização não especificada) seguido dos códigos U07.1 (covid-19, vírus identificado) ou U07.2 (covid-19, vírus não identificado, clínico-epidemiológico) que foram os marcadores da pandemia no Brasil, definidos pela Organização Mundial da Saúde, na parte 1 da declaração de óbito, independentemente da sua posição na cadeia de causas. 

Na tentativa de superar a subestimação dos dados populacionais referentes aos trabalhadores da saúde, foram utilizadas diferentes fontes de informação para o cálculo dos coeficientes de mortalidade. Os dados foram provenientes dos conselhos profissionais, do Cadastro Nacional de Estabelecimentos de Saúde (http://cnes.datasus.gov.br/) e do Sistema de Informações do Programa Nacional de Imunizações (https://opendatasus.saude.gov.br/dataset/covid-19-vacinacao). Para este último, foi considerado o número de trabalhadores da saúde da Bahia vacinados com a primeira dose contra a covid-19. 

Para o cálculo do coeficiente de mortalidade, no numerador foi utilizado o número de óbitos por covid-19 em trabalhadores da saúde. No denominador, para quantificar a população exposta ao risco de morrer por essa doença, foram utilizadas, quando disponíveis, as populações informadas pelas fontes de dados utilizadas neste estudo. Realizou-se a comparação entre essas três fontes de informação populacional para verificar a consistência desse indicador. Os valores calculados para o coeficiente de mortalidade foram apresentados por 1 mil trabalhadores.

Para verificar o efeito das mortes ocorridas precocemente em relação à duração da vida para a população do estudo, foram estimados os anos potenciais de vida perdidos e anos produtivos de vida perdidos por covid-19, por indivíduo, em cada grupo etário. Utilizou-se a fórmula matemática proposta por Romeder e McWhinnie (17), adotando 70 anos no limite superior da idade.

Subtraiu-se a idade em que o trabalhador faleceu por covid-19 do limite superior de idade adotado (representando a expectativa de vida) para estimar os anos potenciais de vida perdidos. O resultado foi multiplicado pelo número de óbitos por covid-19 ocorridos na mesma faixa etária. Realizou-se o somatório dos valores obtidos para todos os trabalhadores que faleceram em cada faixa etária.

Para o cálculo dos anos produtivos de vida perdidos, foi considerado o ponto médio do intervalo de classe das faixas etárias acrescido de fator de correção de 0,5 (empregado quando se arbitra que todas as mortes ocorreram no meio do ano). Esse valor foi subtraído do limite superior da idade adotado como ponto de corte. O resultado dessa subtração foi multiplicado pelo número de óbitos por covid-19 ocorridos na mesma faixa etária. Os valores obtidos foram somados para cada faixa etária, gerando o total dos anos produtivos de vida perdidos.

Analisaram-se os coeficientes de mortalidade por covid-19 e a proporção de vacinados contra a doença em trabalhadores da saúde da Bahia, por quadrimestre, a fim de verificar a tendência da curva de mortalidade. Para o cálculo da proporção de vacinados, no numerador utilizou-se o número de trabalhadores da saúde vacinados no período e, no denominador, a média por quadrimestre de trabalhadores da saúde inseridos no Cadastro Nacional de Estabelecimentos de Saúde.

### Métodos estatísticos 

Foram utilizadas as frequências absolutas e relativas para as variáveis categóricas. A média e o desvio-padrão foram calculados para as variáveis contínuas. Os dados foram tabulados no Microsoft Excel 16.0 e analisados no SPSS 20.0. 

## Resultados

No período analisado, foram notificados 17.605 óbitos por covid-19 na Bahia, destes 4.081 (23,2%) não tinham ocupação preenchida, 2.247 (12,8%) eram aposentados e pensionistas, 384 (2,2%) estavam desempregados, 403 (2,3%) eram trabalhadores da saúde e 8.933 (50,7%), demais trabalhadores. 

Do total de trabalhadores da saúde que foram a óbito por covid-19, a maior parte era do sexo feminino (63,3%), com idade igual ou superior a 40 anos (85,4%), da raça/cor da pele parda (52,1%) e com ensino médio e técnico (48,1%) (Tabela 1). A média de idade foi 53,2 anos (desvio-padrão=10,9 anos).

**Tabela 1:** Características sociodemográficas dos trabalhadores da saúde que foram a óbito por covid-19. Bahia, 2020-2022 (n=403)

Variáveis	n (%)
Sexo	
Feminino	255 (63,3)
Masculino	148 (36,7)
**Faixa etária** (anos)	
19-29	6 (1,5)
30-39	53 (13,1)
40-49	94 (23,3)
50-59	105 (26,1)
60-69	145 (36,0)
**Raça/cor da pele**	
Branca	112 (27,8)
Preta	68 (16,9)
Parda	210 (52,1)
Ignorado/em banco	13 (3,2)
Escolaridade	
Baixa escolaridade	51 (12,7)
Nível médio e técnico	194 (48,1)
Nível universitário	143 (35,5)
Ignorado/em branco	15 (3,7)

As ocupações com maior número de óbitos foram técnicos/auxiliares de enfermagem (29,0%), agentes de saúde (13,4%) e cuidadores de idosos/em saúde (10,4%). O coeficiente geral de mortalidade por covid-19 entre os trabalhadores da saúde na Bahia foi similar entre as três fontes de informação utilizadas para compor a população exposta a morrer por covid-19. Observou-se discrepância entre os coeficientes de mortalidade por ocupação, mas houve concordância, entre as três fontes de informação, em relação às ocupações veterinários/zootecnistas e biólogos ao apresentarem um dos maiores coeficientes de mortalidade.

Os coeficientes de mortalidade, calculados com base nos dados populacionais do Cadastro Nacional de Estabelecimentos de Saúde (CNES) e do Sistema de Informações do Programa Nacional de Imunizações (SI-PNI), apresentaram concordância quanto aos maiores valores observados entre cuidadores de idosos/em saúde, mas divergiram em relação aos agentes de saúde e atendentes de farmácia/consultório (Tabela 2).

**Tabela 2:** Óbitos, população extraída do Cadastro Nacional de Estabelecimentos de Saúde (CNES), conselho e Sistema de Informações do Programa Nacional de Imunizações (SI-PNI), e coeficiente de mortalidade por covid-19, segundo a ocupação, em trabalhadores da saúde. Bahia, 2020-2022 (n=403)

Ocupações	Óbitos	População			Coeficiente de mortalidade		
	n (%)	CNES	Conselho	SI-PNI	CNES	Conselho	SI-PNI
Agentes de saúde	54 (13,4)	39.348	–	1.931	1,4	–	28,0
Assistentes sociais	13 (3,2)	2.759	18.499	4.298	4,7	0,7	3,0
Atendentes de farmácia/ consultório	29 (7,2)	1.990	–	–	14,6	–	–
Biólogos	4 (1,0)	242	3.260	349	16,5	1,2	11,5
Biomédicos	0 (0,0)	851	–	2.071	–	–	–
Cirurgiões dentistas	12 (3,0)	9.673	15.520	11.809	1,2	0,8	1,0
Condutores de ambulância	0 (0,0)	2.382	–	12.497	0,0	–	–
Cuidadores de idosos/em saúde	42 (10,4)	236	–	8.284	178,0	–	5,1
Educadores físicos	4 (1,0)	608	–	2.611	6,6	–	1,5
Enfermeiros	31 (7,7)	23.066	39.675	32.766	1,3	0,8	0,9
Farmacêuticos	10 (2,5)	3.817	11.045	8.828	2,6	0,9	1,1
Fisioterapeutas/quiropraxistas	6 (1,5)	6.468	17.163	12.672	0,9	0,3	0,5
Fonoaudiólogos	0 (0,0)	1.089	1.850	1.332	–	–	–
Gestores de serviços de saúde	1 (0,2)	2.229	–	–	0,4	–	–
Médicos	30 (7,4)	33.542	26.192	21.508	0,9	1,1	1,4
Nutricionistas	1 (0,2)	2.585	10.446	5.739	0,4	0,1	0,2
Outros^a^	9 (2,2)	306	–	1.676	29,4	–	5,4
Psicólogos/psicanalistas	14 (3,5)	3.831	17.696	7.478	3,7	0,8	1,9
Técnicos/ auxiliares de enfermagem	117 (29,0)	47.870	99.200	62.948	2,4	1,2	1,9
Técnicos/ auxiliares de laboratórios de saúde/bancos de sangue	12 (3,0)	4.068	–	–	2,9	–	–
Técnicos/ auxiliares em saúde bucal	5 (1,2)	4.745	–	4.423	1,1	–	1,1
Tecnólogos/ técnicos em radiologia	1 (0,2)	2.689	–	–	0,4	–	–
Terapeutas ocupacionais/ ortoptistas	1 (0,2)	370	624	425	2,7	1,6	2,4
Veterinários/ zootecnistas	7 (1,7)	264	5.590	1.968	26,5	1,3	3,6
Total	403 (100,0)	195.026	266.760	205.613	2,1	1,5	2,0

^a^Doulas, esteticistas, massoterapeutas, podólogos, técnicos em acupuntura, técnicos em quiropraxia, terapeutas holísticos, técnicos em óptica e optometria, técnicos em manutenção de equipamentos e instrumentos médico-hospitalares, técnicos de ortopedia, técnicos de imobilização ortopédica e técnicos em nutrição e dietética.

Na evolução da mortalidade por covid-19 em trabalhadores da saúde, houve aumento da mortalidade no segundo quadrimestre de 2020. Posteriormente, houve redução da mortalidade, a qual voltou a subir no primeiro quadrimestre de 2021. A partir do segundo quadrimestre de 2021, ocorreu queda acentuada da mortalidade por covid-19. Observou-se alta concentração de trabalhadores vacinados com a primeira dose da vacina contra a covid-19 no primeiro quadrimestre de 2021, reduzindo nos meses seguintes (Figura 1).

**Figura 1:** Coeficiente de mortalidade e proporção de vacinados por covid-19 em trabalhadores da saúde, por quadrimestre. Bahia, 2020-2022

Foram estimados 6.771,0 anos potenciais de vida perdidos e 6.778,0 anos produtivos de vida perdidos, dos quais 4.220,0 anos potenciais de vida perdidos e 4.186,0 anos produtivos de vida perdidos foram atribuídos ao sexo feminino, correspondendo a 62,3% e 61,8% da perda. A perda foi maior no sexo feminino em todas as faixas etárias, exceto entre 19-29 anos. O maior impacto no número dos anos potenciais de vida perdidos e anos produtivos de vida perdidos foi observado na faixa etária de 40-49 anos, totalizando 34,2% (2.317,0 anos potenciais de vida perdidos) e 34,7% (2.350,0 anos produtivos de vida perdidos) da perda. O menor impacto foi observado na faixa etária de 19-29 anos (263,0 anos potenciais de vida perdidos; 273,0 anos produtivos de vida perdidos). O ano de 2021 representou perda de 56,2% (3.804,0 anos potenciais de vida perdidos) e 55,9% (3.786,5 anos produtivos de vida perdidos) (Tabela 3). Em relação à média de anos potenciais de vida perdidos/óbito, cada mulher falecida perdeu 16,5 anos e cada homem, 17,2 anos. 

**Tabela 3:** Anos potenciais de vida perdidos (APVP) e anos produtivos de vida perdidos (APrVP) por covid-19 em trabalhadores da saúde, por faixa etária e sexo. Bahia, 2020-2022 (n=403)

Faixa etária (anos)	Sexo	2020		2021		2022		Total	
		APVP	APrVP	APVP	APrVP	APVP	APrVP	APVP	APrVP
19-29	Feminino	43,0	45,5	0,0	0,0	51,0	45,5	94,0	91,0
	Masculino	0,0	0,0	126,0	136,5	43,0	45,5	169,0	182,0
	Total	43,0	45,5	126,0	136,5	94,0	91,0	263,0	273,0
30-39	Feminino	340,0	350,0	580,0	595,0	136,0	140,0	1.056,0	1.085,0
	Masculino	405,0	420,0	344,0	350,0	0,0	0,0	749,0	770,0
	Total	745,0	770,0	924,0	945,0	136,0	140,0	1.805,0	1.855,0
40-49	Feminino	493,0	500,0	964,0	975,0	46,0	50,0	1.503,0	1.525,0
	Masculino	239,0	250,0	520,0	525,0	55,0	50,0	814,0	825,0
	Total	732,0	750,0	1.484,0	1.500,0	101,0	100,0	2.317,0	2.350,0
50-59	Feminino	499,0	480,0	476,0	480,0	68,0	60,0	1.043,0	1.020,0
	Masculino	229,0	240,0	295,0	300,0	15,0	15,0	539,0	555,0
	Total	728,0	720,0	771,0	780,0	83,0	75,0	1.582,0	1.575,0
60-69	Feminino	133,0	125,0	312,0	270,0	79,0	70,0	524,0	465,0
	Masculino	57,0	70,0	187,0	155,0	36,0	35,0	280,0	260,0
	Total	190,0	195,0	499,0	425,0	115,0	105,0	804,0	725,0
Total	Feminino	1.508,0	1.500,5	2.332,0	2.320,0	380,0	365,5	4.220,0	4.186,0
	Masculino	930,0	980,0	1.472,0	1.466,5	149,0	145,5	2.551,0	2.592,0
	Total	2.438,0	2.480,5	3.804,0	3.786,5	529,0	511,0	6.771,0	6.778,0

As ocupações que apresentaram as maiores perdas foram aquelas que atuaram diretamente no cuidado assistencial, ou seja, que estavam mais expostas ao adoecimento pela covid-19 (Tabela 4). Os trabalhadores da saúde acometidos precocemente, entre 19-29 anos, foram aqueles com ensino médio e técnico, cirurgiões dentistas, educadores físicos e psicólogos/psicanalistas. Na faixa etária de 40-49 anos, as ocupações com maior perda foram trabalhadores com ensino médio e técnico, enfermeiros e médicos (Tabela 4).

**Tabela 4:** Anos potenciais de vida perdidos (APVP) e anos produtivos de vida perdidos (APrVP) por covid-19 em trabalhadores da saúde, segundo ocupação e faixa etária. Bahia, 2020-2022 (n=403)

Ocupação	19-29 anos		30-39 anos		40-49 anos		50-59 anos		60-69 anos		Total	
	APVP	APrVP	APVP	APrVP	APVP	APrVP	APVP	APrVP	APVP	APrVP	APVP	APrVP
Agentes de saúde	0,0	0,0	229,0	245,0	317,0	325,0	222,0	225,0	117,0	95,0	885,0	890,0
Assistentes sociais	0,0	0,0	32,0	35,0	45,0	50,0	60,0	60,0	42,0	30,0	179,0	175,0
Atendentes de farmácia/consultório	43,0	45,5	110,0	105,0	121,0	125,0	200,0	210,0	44,0	30,0	518,0	515,5
Biólogos	0,0	0,0	0,0	0,0	51,0	50,0	0,0	0,0	8,0	10,0	59,0	60,0
Cirurgiões dentistas	43,0	45,5	77,0	70,0	22,0	25,0	60,0	60,0	7,0	20,0	209,0	220,5
Cuidadores de idosos/em saúde	0,0	0,0	0,0	0,0	393,0	400,0	245,0	225,0	74,0	55,0	712,0	680,0
Educadores físicos	42,0	45,5	35,0	35,0	47,0	50,0	0,0	0,0	0,0	0,0	124,0	130,5
Enfermeiros	0,0	0,0	301,0	315,0	206,0	200,0	94,0	90,0	38,0	40,0	639,0	645,0
Farmacêuticos	0,0	0,0	66,0	70,0	46,0	50,0	50,0	45,0	13,0	15,0	175,0	180,0
Fisioterapeutas/quiropraxista	0,0	0,0	141,0	140,0	0,0	0,0	0,0	0,0	15,0	10,0	156,0	150,0
Gestores de serviços de saúde	0,0	0,0	0,0	0,0	0,0	0,0	16,0	15,0	0,0	0,0	16,0	15,0
Médicos	0,0	0,0	0,0	0,0	101,0	100,0	127,0	135,0	88,0	85,0	316,0	320,0
Nutricionistas	0,0	0,0	0,0	0,0	0,0	0,0	20,0	15,0	0,0	0,0	20,0	15,0
Outros^a^	0,0	0,0	101,0	105,0	72,0	75,0	0,0	0,0	14,0	15,0	187,0	195,0
Psicólogos/psicanalistas	41,0	45,5	101,0	105,0	79,0	75,0	17,0	15,0	16,0	30,0	254,0	270,5
Técnicos/auxiliares de enfermagem	43,0	45,5	442,0	455,0	695,0	700,0	421,0	420,0	255,0	235,0	1.856,0	1.855,5
Técnicos/auxiliares de laboratórios de saúde/banco de sangue	0,0	0,0	135,0	140,0	27,0	25,0	11,0	15,0	43,0	30,0	216,0	210,0
Técnicos/auxiliares em saúde bucal	51,0	45,5	0,0	0,0	47,0	50,0	15,0	15,0	9,0	5,0	122,0	115,5
Tecnólogos/técnicos em radiologia	0,0	0,0	0,0	0,0	0,0	0,0	12,0	15,0	0,0	0,0	12,0	15,0
Terapeutas ocupacionais/ortoptistas	0,0	0,0	0,0	0,0	0,0	0,0	0,0	0,0	9,0	5,0	9,0	5,0
Veterinários/zootecnistas	0,0	0,0	35,0	35,0	48,0	50,0	12,0	15,0	12,0	15,0	107,0	115,0
Total	263,0	273,0	1.805,0	1.855,0	2.317,0	2.350,0	1.582,0	1.575,0	804,0	725,0	6.771,0	6.778,0

^a^Pedólogos, técnicos em óptica, técnicos em optometria, técnicos de ortopedia, esteticistas e massagistas.

## Discussão

Esta pesquisa estimou o efeito da mortalidade por covid-19 em diferentes grupos de trabalhadores da saúde na Bahia. Observou-se maior impacto no sexo feminino, na faixa etária de 40-49 anos, entre os pardos, com ensino médio e técnico, e nas ocupações de técnicos/auxiliares de enfermagem, agentes de saúde, cuidadores de idosos/em saúde, enfermeiros, atendentes de farmácia/consultório e médicos. O coeficiente geral de mortalidade por covid-19 foi similar entre as três fontes de informação utilizadas, embora tenha havido discrepâncias ao comparar os coeficientes por ocupação. Em relação à evolução da mortalidade, observaram-se três ondas, com magnitude marcadamente distinta no último ano. O início da vacinação no primeiro quadrimestre de 2021 resultou na redução drástica da mortalidade entre trabalhadores da saúde.

O sexo masculino, a idade avançada e a comorbidade estão associados à maior ocorrência de óbitos por covid-19 em trabalhadores da saúde como em não trabalhadores da saúde (18-21). No presente estudo, observou-se maior número de óbitos no sexo feminino. Isso se deve, possivelmente, à maior proporção de mulheres no setor saúde, haja vista que não foi possível o cálculo do coeficiente de mortalidade por sexo, pois não houve dados populacionais disponíveis em trabalhadores da saúde. A maior proporção entre os óbitos foi crescente com a idade. 

Os resultados deste estudo chamam atenção para as ocupações de cuidadores de idosos/em saúde, veterinários/zootecnistas, biólogos e atendentes de farmácia/consultório, pois tiveram os maiores coeficientes de mortalidade e até 2024 não havia relatos na literatura sobre mortalidade por covid-19 nessas ocupações. Considerando a fragilidade da variável ocupação, mesmo no registro de óbitos, e as profundas divergências dos denominadores, esses achados devem ser destacados, mas a força da evidência deve ser tomada com a parcimônia necessária.

No México, evidenciou-se que as pessoas que trabalhavam em ambulância (44,8/10 mil trabalhadores), fisioterapeutas (22,4/10 mil trabalhadores) e equipes de farmácia (20,7/10 mil trabalhadores) apresentaram os maiores coeficientes de mortalidade (21). Apesar da pandemia da covid-19 apresentar estabilidade e redução da gravidade e mortalidade, ainda são escassos na literatura os estudos que calcularam o coeficiente de mortalidade entre trabalhadores da saúde, possivelmente devido à dificuldade em obter dados sobre a população exposta ao risco de morrer pela doença.

A mortalidade por covid-19 evoluiu em três ondas, com maior pico na segunda, e os menores coeficientes registrados na terceira (9), corroborando os achados deste estudo. A evolução da mortalidade na segunda onda pode ser explicada pelo surgimento da nova variante Delta (22), que coincidiu com o início da vacinação. As campanhas de vacinação reduziram a morbimortalidade por covid-19 (23,24). 

Através do levantamento dos óbitos, foi possível estimar indicadores de impacto, que revelaram o valor social da mortalidade prematura e a redução da força de trabalho. Os achados deste estudo demonstraram maior impacto em trabalhadores jovens na faixa etária entre 40-49 anos. Quando a morte ocorre em uma fase em que a vida é potencialmente produtiva, ela atinge não somente o indivíduo, mas também a sociedade como um todo, pois perde-se o seu potencial econômico e intelectual (25,26). 

Os anos potenciais de vida perdidos são um indicador que mede quanto tempo uma pessoa teria vivido se não tivesse morrido prematuramente. Esse indicador permite identificar as faixas etárias mais afetadas pela doença, mostrando-se eficiente na priorização de intervenções em saúde pública (25-27).

Até 2024, não havia estudos com o mesmo escopo metodológico sobre anos potenciais de vida perdidos e anos produtivos de vida perdidos por covid-19 em trabalhadores da saúde, o que impossibilitou realizar comparação. No Brasil, os anos de vida perdidos por morte prematura devido à covid-19 foram maiores entre os enfermeiros e técnicos de enfermagem, do sexo masculino, na faixa etária de 41-50 anos (28). Também se observou maior perda entre os profissionais da enfermagem na faixa etária de 31-40 anos, sendo menor entre os mais jovens (29). 

Houve grande impacto nos anos potenciais de vida perdidos devido à covid-19, considerando a raça/cor da pele e o gênero. Observou-se maior perda entre os homens, e as populações negra e indígena foram mais atingidas em faixas etárias mais jovens, em comparação às populações branca e amarela (30).

Este estudo apresentou limitações que devem ser consideradas na interpretação dos resultados. Em primeiro lugar, foram utilizados dados secundários que podem interferir na validade dos achados, com viés de seleção e informação, sobretudo pela incompletude e subnotificação dos dados, com inexistência de dados sobre algumas ocupações. Em segundo lugar, houve dados populacionais subestimados e ausência de dados estratificados por sexo e idade para compor o denominador. Em terceiro lugar, foi utilizado um único limite superior da idade de 70 anos para calcular os anos potenciais de vida perdidos e anos produtivos de vida perdidos. Essa abordagem não levou em consideração que a expectativa de vida varia com o sexo, o que pode levar à superestimação da medida para o sexo masculino, tendo em vista que a expectativa de vida no sexo masculino é inferior ao sexo feminino. 

Os pontos fortes deste estudo incluíram a diversidade de categorias de trabalhadores da saúde estudadas e a estratégia adotada para a superação da ausência de um único registro populacional de trabalhadores da saúde com a busca de três fontes: Cadastro Nacional de Estabelecimentos de Saúde, conselhos profissionais e Sistema de Informações do Programa Nacional de Imunizações. Essa iniciativa permitiu o cálculo de coeficientes de mortalidade que não têm sido realizados na maioria dos estudos identificados com trabalhadores da saúde. Destacou o ineditismo em quantificar anos potenciais de vida perdidos e anos produtivos de vida perdidos por covid-19 em trabalhadores da saúde. 

Os resultados deste estudo mostraram potencial impacto gerado pela covid-19 na força de trabalhadores da saúde da Bahia, principalmente em grupos de trabalhadores pouco mencionados na literatura, ainda que sejam vinculados a conselhos profissionais. Os resultados trouxeram contribuições que podem subsidiar estratégias de intervenções no campo da saúde do trabalhador e fornecer informações sobre o impacto da covid-19 em trabalhadores da saúde; servir de aprendizado para novas emergências de saúde pública; e fomentar políticas públicas de enfretamento de epidemias de doenças respiratórias, como o programa de vacinação e as ações voltadas para a promoção da saúde do trabalhador. 

Apesar dos esforços de compreender o comportamento da covid-19, ainda existem custos sociais e econômicos desconhecidos. Dessa forma, é necessária a realização de novos estudos sobre a magnitude da mortalidade precoce em trabalhadores da saúde no contexto da pandemia da covid-19. 

## Data Availability

As bases de dados utilizadas neste estudo são de acesso público, e os links para sua obtenção estão detalhados nos métodos. Os dados tabulados estão disponíveis em: https://demo.dataverse.org/dataset.xhtml?persistentId=doi%3A10.70122%2FFK2%2FSYHSJL&version=DRAFT.
